# Supplementary material for: A seq2seq model to forecast the COVID-19 cases, deaths and reproductive R numbers in US counties
Source: Res Sq. 2021 Apr 26:rs.3.rs-456641. Preprint. [Version 1] doi: 10.21203/rs.3.rs-456641/v1 (PMC8132245; doi:10.21203/rs.3.rs-456641/v1)
Supplement: Supplement 1 [file f3556b26fd502c75e92b1742.docx]

**Supplementary Materials**

**Title: A seq2seq model to forecast the COVID-19 cases, deaths and reproductive *R* numbers in US counties**

**Authors:** Yanli Zhang-James^1^, Jonathan Hess^1^, Asif Salekin^2^, Dongliang Wang^3^, Samuel Chen^4^, Peter Winkelstein^5^, Christopher P Morley^3,6^ and Stephen V Faraone^1,7^

**Affiliations:**

1. Department of Psychiatry and Behavioral Sciences, SUNY Upstate Medical University, Syracuse, New York, USA
2. Department of Electrical Engineering and Computer Science, Syracuse University, Syracuse, New York, USA
3. Department of Public Health & Preventive Medicine, SUNY Upstate Medical University, Syracuse, New York, USA
4. School of Medicine, SUNY Upstate Medical University, Syracuse, New York, USA
5. Institute for Healthcare Informatics, University at Buffalo, Buffalo, New York, USA.
6. Department of Family Medicine, SUNY Upstate Medical University, Syracuse, New York, USA
7. Department of Neuroscience and Physiology, SUNY Upstate Medical University, Syracuse, New York, USA

**Corresponding author:**

Yanli Zhang-James, MD PHD

Research Associate Professor

Department of Psychiatry and Behavioral Sciences

SUNY Upstate Medical University

3732B Neuroscience Research Building

505 Irving Ave. Syracuse, NY 13210

Phone: 315-464-3441

Email: Zhangy@upstate.edu

Supplementary Figure 1. ML setup.

a Model selection, tuning and testing process


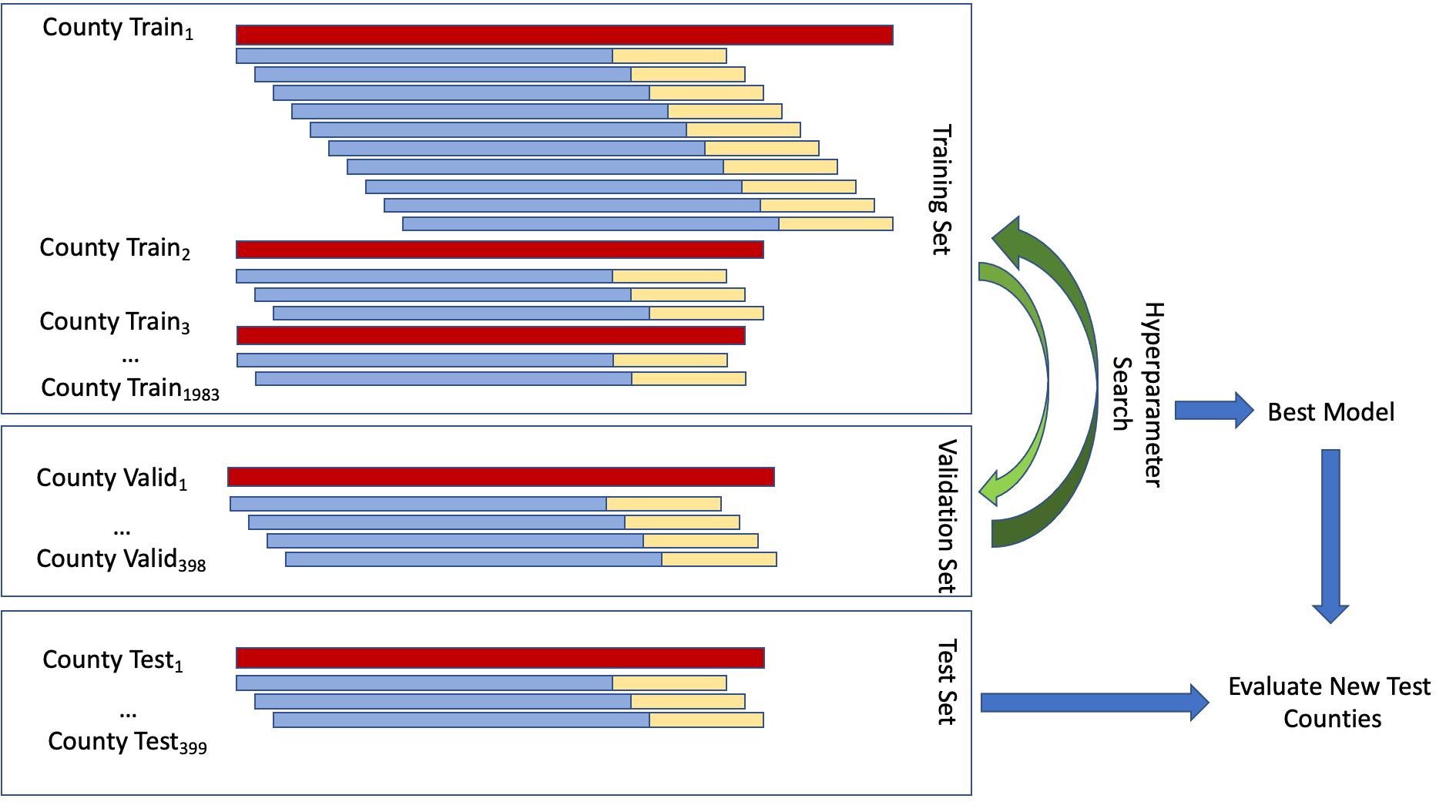


b Making Forecast.


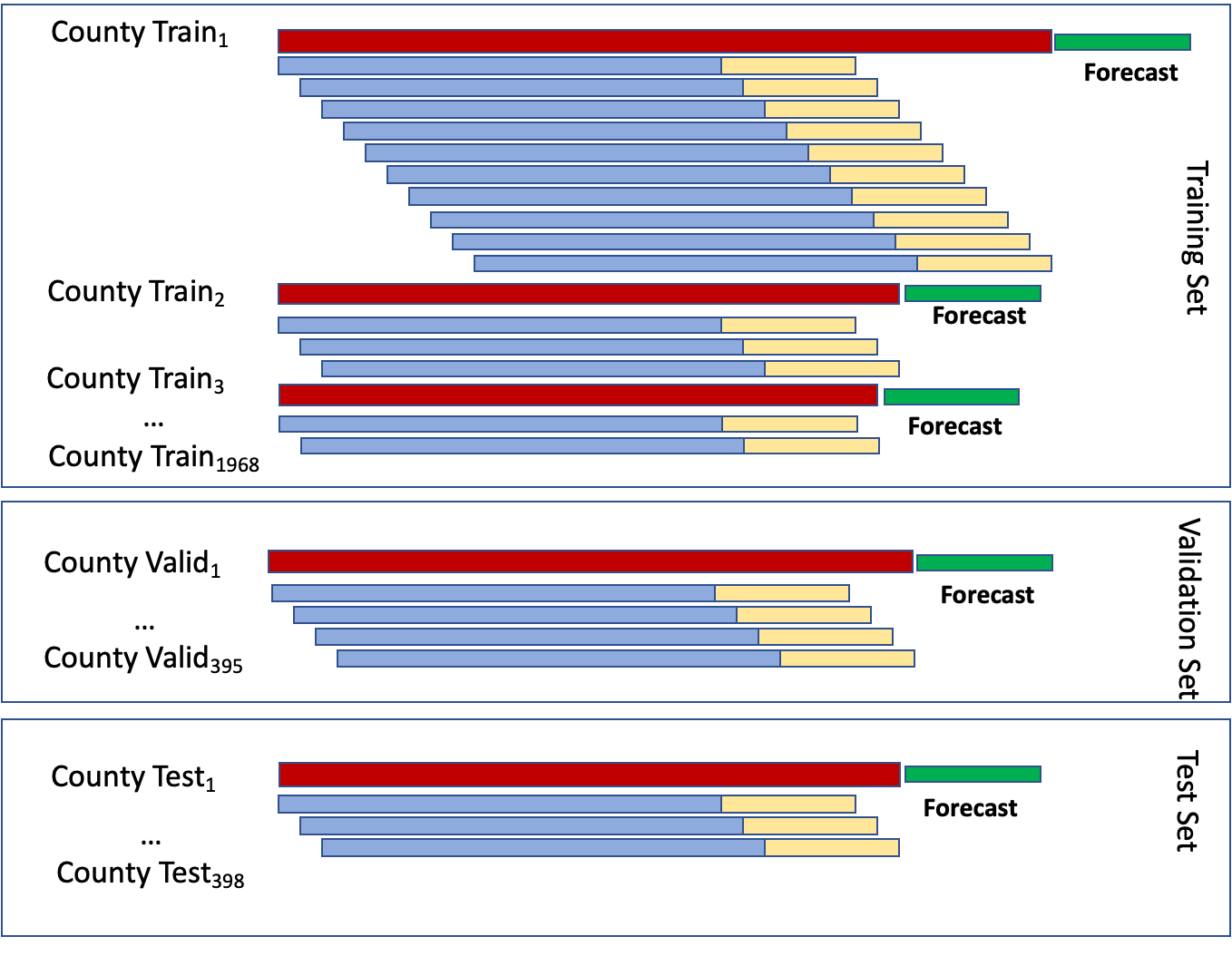


Supplementary Figure 2. HyperOpt results of various input lenghth.


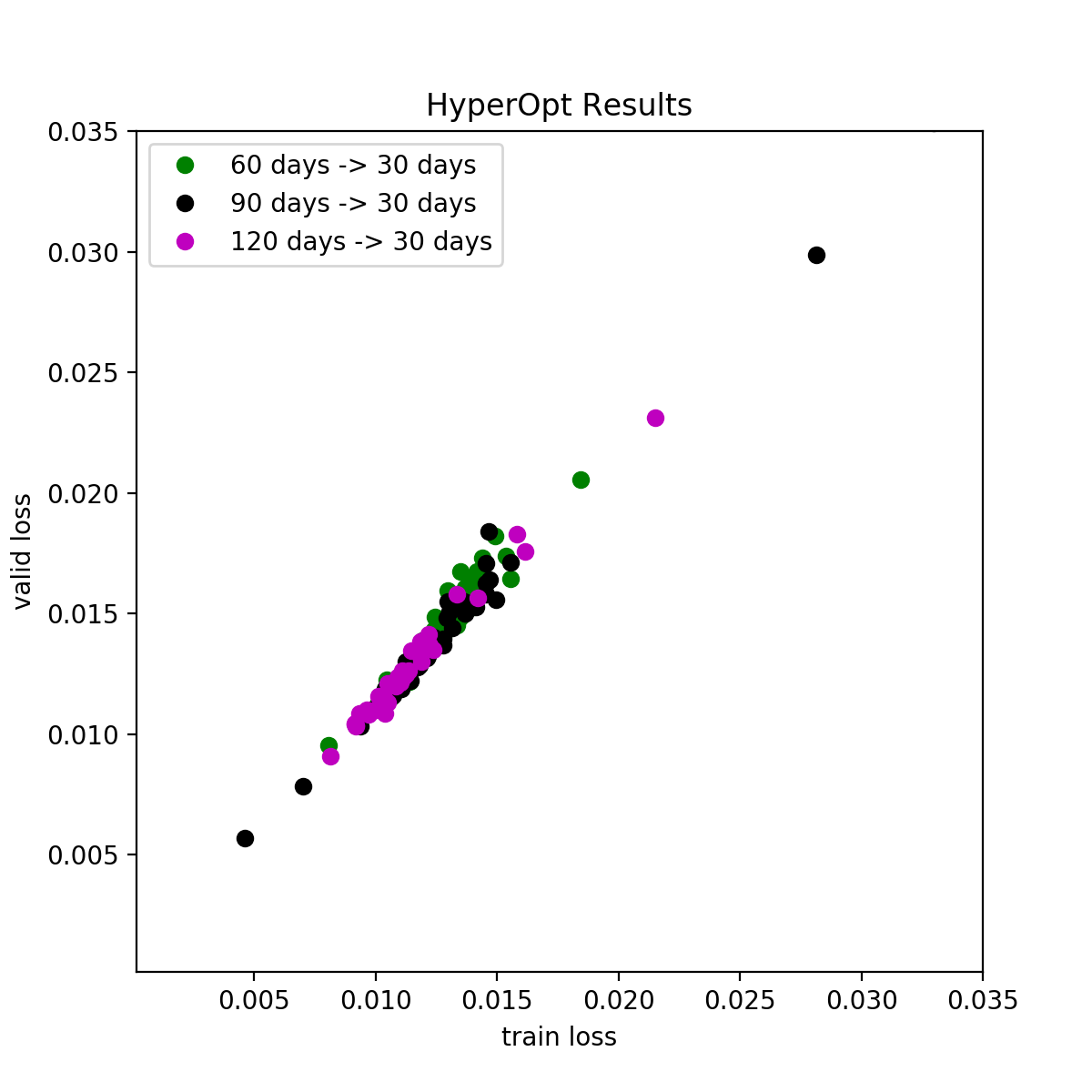


Supplementary Figure 3 a Daily predicted cases (y axis) vs actual cases (x axis) over the next 30 days from the forecast date Nov 30^th^, 2020. b Daily predicted deaths (y axis) vs actual deaths (x axis) over the 30 days forecast. c Daily predicted R values (y axis) vs R calculated from the actual cases (x axis) over the 30 days forecast. d Relative Error distributions.

a


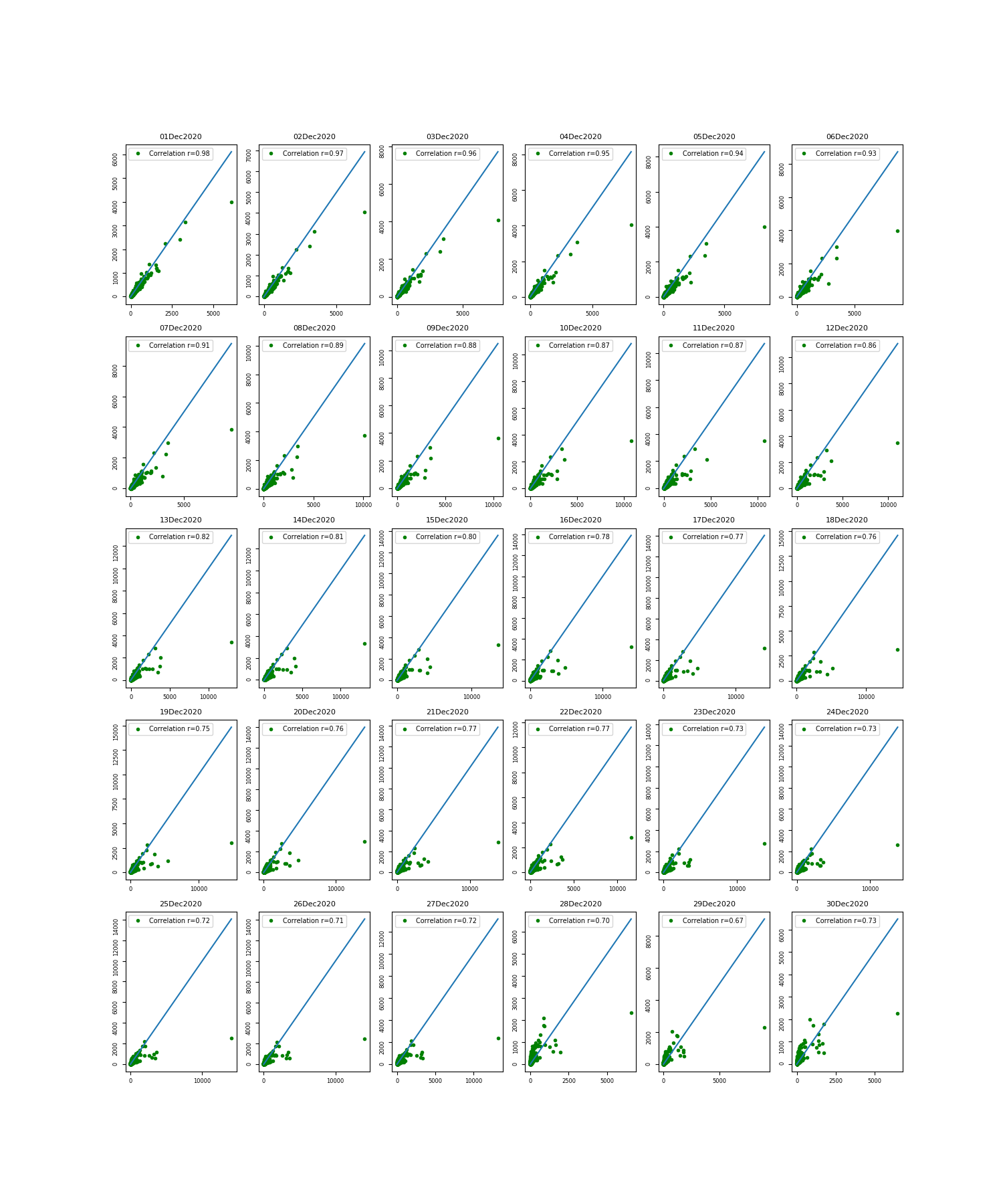


b


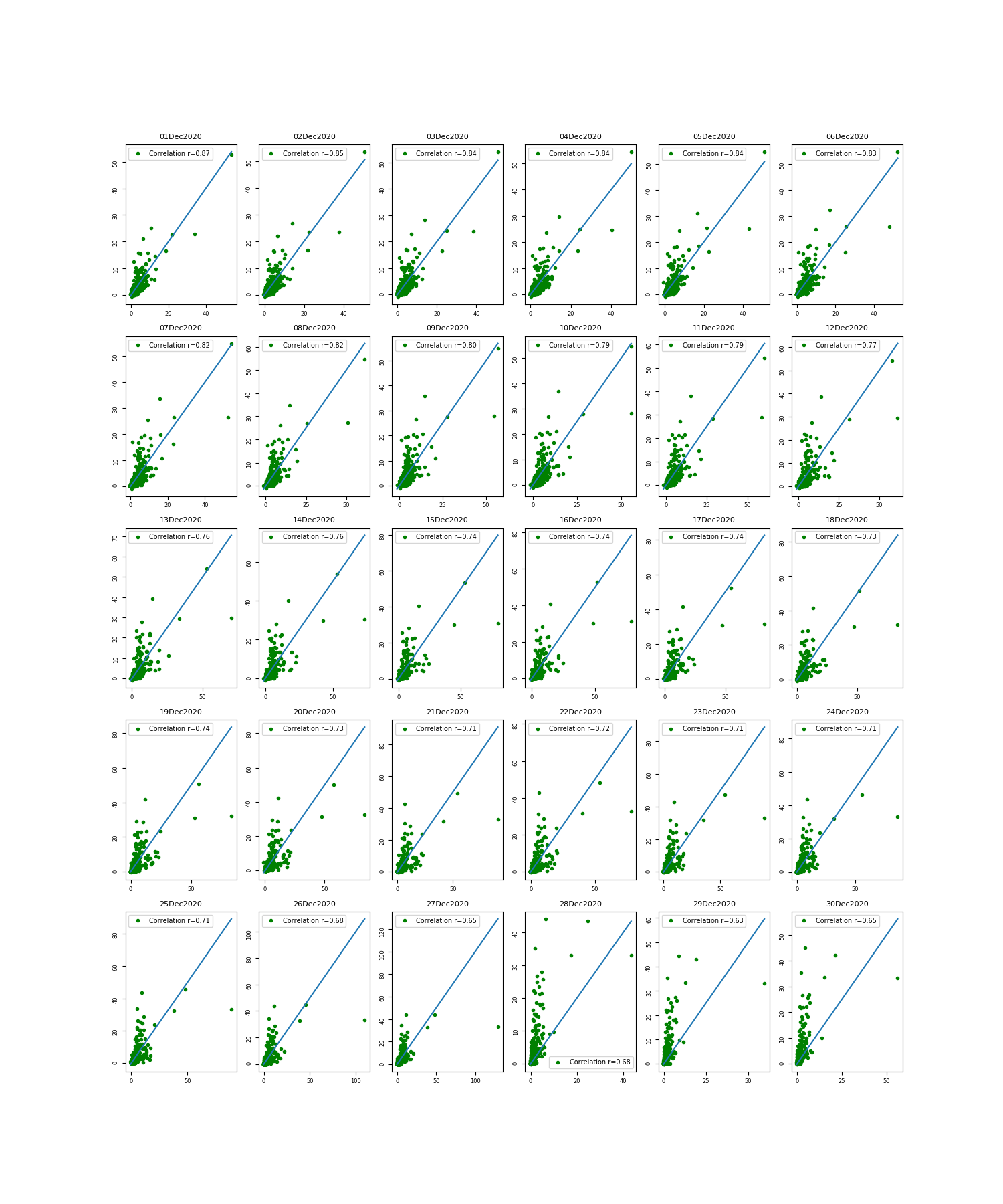


c


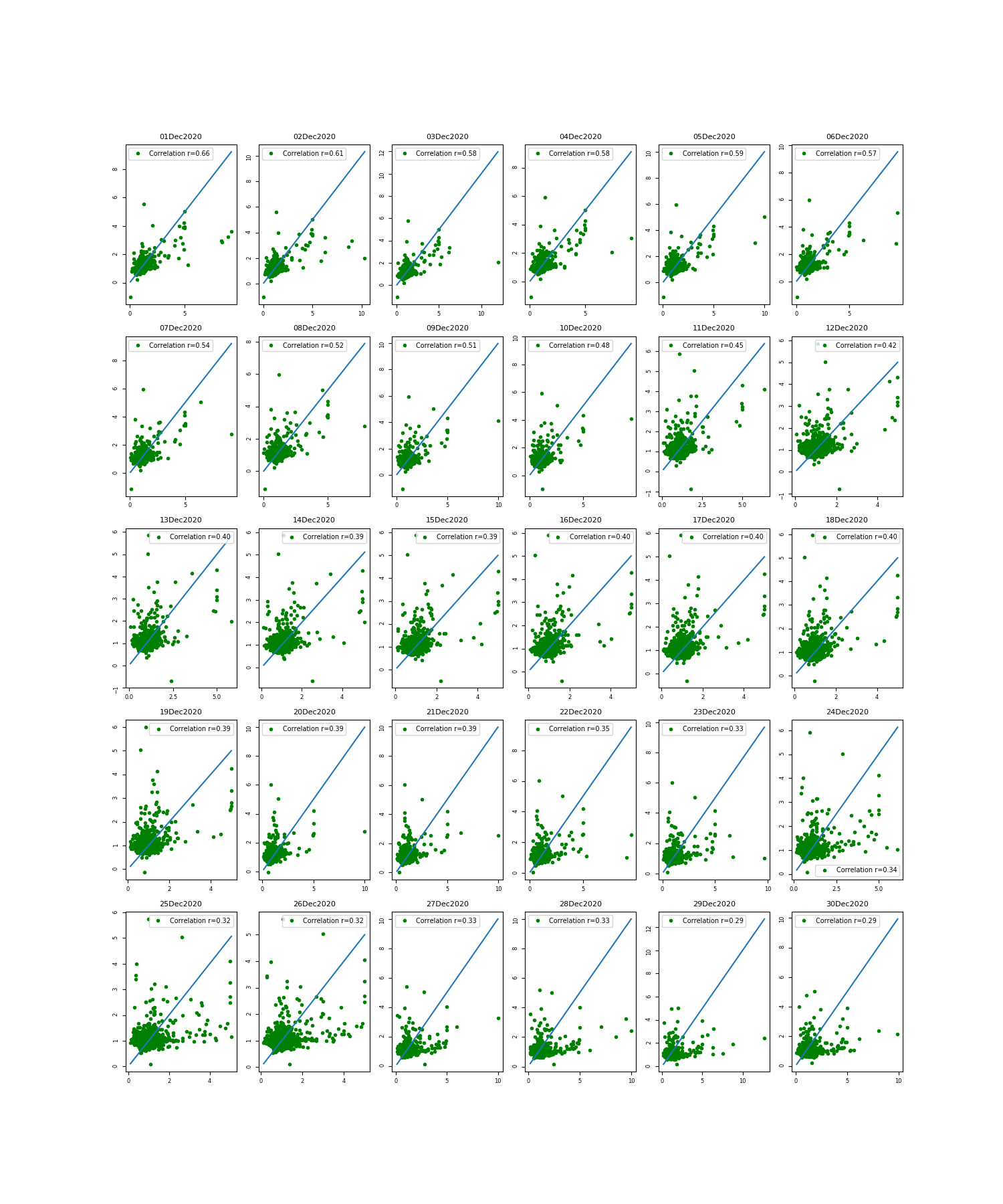


d


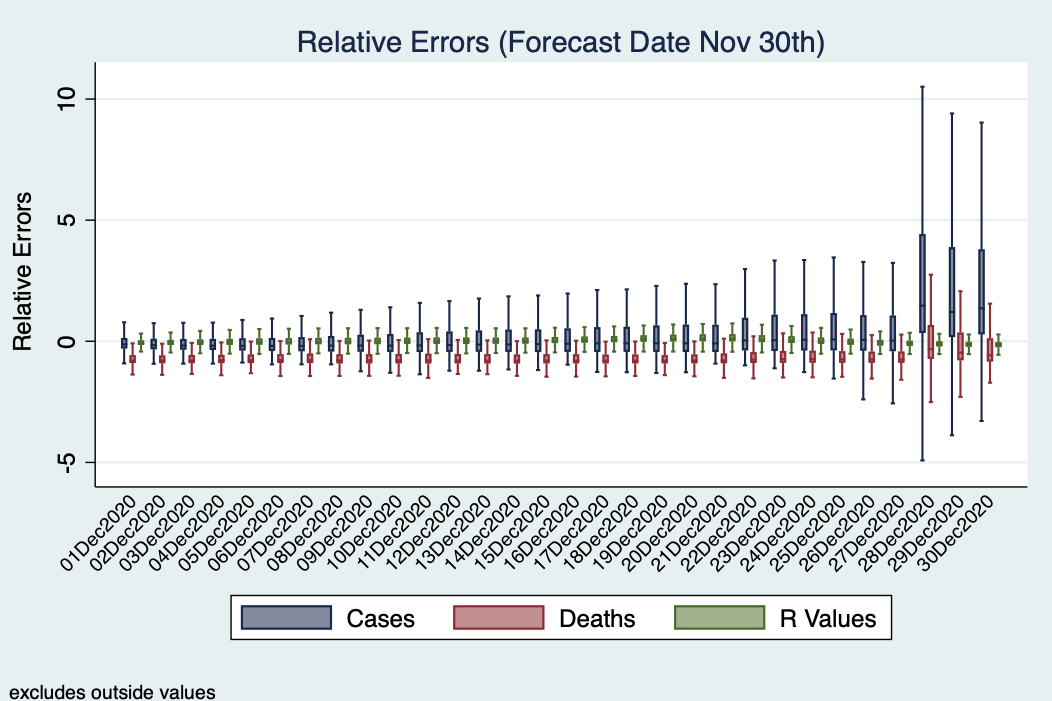


Supplementary Table 1. Median relative errors for county-level case forecast from different teams.

|  | Forecast Date Nov 23, 2020 | | | | | Forecast Date Nov 30, 2020 | | | | |
| --- | --- | --- | --- | --- | --- | --- | --- | --- | --- | --- |
|  | Numbers of Counties Predicted | Median REs* | | | | Numbers of Counties Predicted | Median REs | | | |
| Team Name |  | Week 1 | Week 2 | Week 3 | Week 4 |  | Week 1 | Week 2 | Week 3 | Week 4 |
| CMU | 188 | 17.8% | 19.4% | 35.4% | 37.5% | 188 | -6.3% | 2.5% | 25.1% | 51.5% |
| Columbia | 2743 | -1.7% | -15.2% | -21.4% | -22.9% | 2767 | -17.1% | -26.5% | -25.1% | -12.6% |
| Ensemble | 2769 | 2.2% | -12.7% | -19.0% | -15.2% | 2769 | -18.1% | -24.4% | -21.0% | -4.9% |
| ISU | 2769 | 16.7% | -2.1% | -10.5% | -7.5% | 2769 | 0.0% | -6.7% | -4.2% | 13.3% |
| JHU_APL | 2769 | 1.0% | -5.9% | -5.9% | 6.4% | 2769 | -18.9% | -25.2% | -27.4% | -14.8% |
| JHU_IDD | 2769 | -37.9% | -45.0% | -46.2% | -47.3% | 2769 | -49.3% | -50.0% | -47.3% | -37.0% |
| LANL | 2768 | 6.2% | -0.6% | -5.3% | -7.8% | 2768 | -22.3% | -26.0% | -21.5% | -8.5% |
| LNQ | 2769 | 5.3% | -5.5% | -4.2% | 4.4% | 2769 | -25.0% | -30.3% | -22.7% | -3.7% |
| UCLA | 1233 | -4.7% | -41.6% | -59.9% | -67.0% | 865 | -15.8% | -27.3% | -26.4% | -20.0% |
| UGA_CEID | 2769 | 6.3% | -11.2% | -18.3% | -14.7% | 2769 | -15.7% | -21.8% | -17.9% | -2.4% |
| UMass_MB | 457 | 10.4% | 2.9% | 1.0% | 18.7% | 457 | -20.2% | -31.6% | -27.3% | -21.0% |
| UVA | 2767 | 7.3% | -10.2% | -13.6% | -9.9% | 2767 | 0.0% | -6.3% | -4.9% | 10.2% |
| UpstateSU | 2769 | -8.9% | -20.7% | -21.2% | -10.5% | 2769 | -11.7% | -14.0% | -10.6% | 4.1% |

*Relative Errors (REs) = (predicted – observed)/observed values.

*This table included all counties that were reported by each team/model.
